# The 7 Pillars of Pain Management for Minimally Invasive Cardiac Surgery

**DOI:** 10.1177/15569845251358225

**Published:** 2025-07-27

**Authors:** Alexander J. Gregory, Christopher D. Noss, William D. T. Kent, Corey Adams, Rakesh C. Arora, Rawn Sallenger

**Affiliations:** 1 Department of Anesthesiology, Perioperative and Pain Medicine, Cumming School of Medicine, University of Calgary, AB, Canada; 2 Libin Cardiovascular Institute, Department of Cardiac Sciences, University of Calgary, AB, Canada; 3 Harrington Heart and Vascular Institute - University Hospitals, Cleveland, OH, USA; 4 Division of Cardiac Surgery, St. Joseph Medical Center, University of Maryland, Towson, MD, USA; 5 Division of Cardiac Surgery, University of Maryland School of Medicine, Baltimore, MD, USA

## Introduction

The foundation for minimally invasive cardiac surgery (MICS) began in the late 1990s when Carpentier and Cosgrove independently published results of their novel approaches to valve surgery without the need for a sternotomy.^1,2^ Over the subsequent decades, there has been steady growth in the use of minimally invasive approaches, including the development of various surgical techniques and evolution of perioperative care.^3–6^ Some centers report increasing MICS use as their programs evolve, with utilization rates approaching 100%.^7,8^ Coronary, mitral, tricuspid, aortic, atrial septal defect, and intracardiac mass removal procedures can now all be performed through a thoracotomy approach.^3,6,9–11^ Although the potential recovery benefits of MICS are well documented, to achieve the best results it is imperative that health care teams appreciate the importance of postoperative analgesia.^12,13^ Lateral thoracotomy incisions, which many MICS procedures rely upon, are thought to be one of the most painful incisions and can create challenges for providing effective pain control.^14–16^ Poorly controlled pain has several undesirable effects that impede postoperative recovery such as anorexia, reduced mobilization, delirium, and sleep deprivation.^17^ Its importance to patient recovery is reflected in it being one of the recommendations of the Enhanced Recovery After Cardiac Surgery (ERAS® Cardiac) and The Society of Thoracic Surgeons 2024 Expert Consensus Statement on perioperative care of cardiac surgical patients.^18^ Through a combination of experience and evidence-based best practices, we propose the following 7 Pillars to optimize analgesia for MICS.

## 1. Build a Multidisciplinary Team

Postoperative pain following MICS is multifactorial and can be mitigated by actions initiated throughout the continuum of care. As with any cardiac surgical quality improvement initiative, building a multidisciplinary team is crucial to successful assessment and treatment of postoperative pain after MICS.^19–21^ Interventions for pain can begin preoperatively with education often provided by the cardiac surgeon and the advanced practice provider setting expectations regarding postoperative pain and the team’s approach to treatment. The cardiac anesthesiologist, with background knowledge of pathophysiology and pharmacology of pain, can provide valuable expertise to achieve optimal management. Postoperatively, critical care nursing is critical for effectively grading pain and assessing the efficacy of treatment, especially for patients who may be limited in communicating for clinical reasons. As physical therapists and other allied health staff help to mobilize patients, they can help ease nonincisional pain as well as provide valuable feedback regarding the limitations postoperative pain may be causing individual patients. The critical care team can assess pain levels on rounds daily and, with the aid of clinical pharmacists and local pain management specialists, adjust individual patient regimens as indicated. The complex nature of pain after MICS can include incisional pain, nonincisional pain, chronic pain, visceral pain, and anxiety.^22^ Different aspects of pain may dominate at various times throughout the postoperative course. Having a multidisciplinary team working together can help tailor a comprehensive pain management strategy specific to each patient’s needs.^20,22–24^

## 2. Block the Intercostal Nerves

The pathophysiology of thoracotomy pain is multifactorial, including nociceptive pathways (both somatic and visceral), neuropathic, and referred.^16^ The intercostal nerve and its lateral cutaneous branch innovate the various muscle layers of the lateral chest wall, which are disrupted by the thoracotomy incision.^14^ Furthermore, there is significant movement between the ribs during breathing (more so than the sternum) that contributes to postoperative pain.^25^ Therefore, we believe that some method of intercostal nerve blockade is an essential component of postoperative analgesia following MICS. One approach is the use of regional anesthesia, particularly chest wall fascial plane blocks. Developed mostly over the past decade, these various blocks share a common use of ultrasound to position a needle, with or without a catheter, to deliver local anesthetic within the fascial planes that house the various branches of the intercostal nerves.^26^ These blocks have been consistently shown to decrease opioid use after thoracotomy, including minithoracotomy for MICS.^19,27^ Another option for intercostal block is through cryoanalgesia, which has been shown to provide improved pulmonary mechanics as well as effective short-term and midterm analgesia for several months following MICS.^28,29^ Finally, if either of these options are not possible, a single injection of local anesthetic under direct visualization by the surgeon has been shown to provide analgesic benefits.^30^ In the end, irrespective of technique, addressing pain at one of its primary sources by blocking the intercostal nerve will provide improved pain control following MICS.

## 3. Optimize Multimodal Opioid-Sparing Analgesia

Blocking the intercostal nerve alone may not be sufficient to provide effective postoperative analgesia. This can be for various reasons including failure of intercostal block technique, incomplete nerve block, or alternative sources of pain not originating through the intercostal nerve pain pathway.^16^ Although opioid-based analgesia had been the traditional approach to postoperative pain management, there has been a recent shift toward a multimodal opioid-sparing approach. This is in part due to the significant constellation of opioid-related side effects (see Pillar 4). But it is also due to concerns over new persistent opioid use, defined as patients not previously on opioids continuing to require them months after surgery, which has previously been shown to occur in approximately 10% of cardiac surgical patients.^31^ There are many analgesic options including acetaminophen, dexmedetomidine, gabapentinoids, glucocorticoids, ketamine, and nonsteroidal anti-inflammatory drugs. Two recent expert consensus documents have reviewed the level of evidence for a variety of multimodal analgesic agents in cardiac surgery patients.^32,33^ The ERAS® Cardiac Society has also recently published an open-access turnkey order set that provides a template for ordering multimodal analgesia medications through all phases of care, including doses, route, and administration instructions.^34^

## 4. Monitor for Side Effects Related to Analgesic Therapies

There are numerous side effects attributed to opioids including respiratory depression, drowsiness, lightheadedness, cognitive dysfunction (including delirium), gastrointestinal dysfunction (nausea, vomiting, and constipation), hypersensitivity to pain, and immune suppression.^17,35^ These side effects can occur in up to 40% of cardiac surgery patients.^36^ Opioids are not alone in their potential for causing adverse effects. In fact, most of the nonopioid multimodal analgesia medications have some potential to have detrimental impact on patients (Table 1). As such, it is important to educate patients, families, and healthcare providers on the possible side effects of all analgesic medications.^37^ Regular monitoring and recording of any side effects will allow for communication within the perioperative team to adjust medication choices and doses to provide the most effective analgesia possible while encountering the least amount of side effects.

**Table 1.** Common Nonopioid Analgesic Medications Side Effects and Adverse Events.

Medication	Potential side effects and adverse events
Acetaminophen	Liver toxicity
Dexamethasone	Hyperglycemia, immune suppression, sleep disturbance
Dexmedetomidine	Bradycardia, hypotension, hypertension, sedation, confusion/agitation, nausea
Gabapentinoids	Sedation, unsteadiness/dizziness, confusion/agitation, sleep disturbance, altered mood, nausea, vision changes, dry mouth
Ketamine	Hallucinations, confusion/agitation, unsteadiness/dizziness, sleep disturbance, nausea, amnesia
Lidocaine	Local anesthetic toxicity, unsteadiness/dizziness, vision changes, nausea, headache
Magnesium	Muscle weakness, hypotension, confusion, unsteadiness/dizziness, flushing, sedation
NSAIDs	Renal injury, GI irritation/bleeding, bleeding, increased CV events
Tramadol	Sedation, unsteadiness/dizziness, constipation, nausea, headache, dry mouth

Abbreviations: CV, cardiovascular; GI, gastrointestinal; NSAIDs, nonsteroidal anti-inflammatory drugs.

## 5. Remove Chest Tubes as Soon as Safely Possible

Chest tubes, although essential early postoperatively, are associated with nociceptive stimulation of the intercostal nerves and visceral irritation of the pleura that may be ineffectively treated with systemic analgesia or intercostal block (even thoracic epidural).^14,16^ Studies have shown that early removal, within the first 24 h after surgery, can significantly reduce pain scores and opioid consumption without increasing the risk of tamponade, pneumothorax, or pleural effusion.^38^ In MICS, where surgical trauma and bleeding is less, the threshold for safe drain removal is lower. A recently published rapid-recovery protocol for patients after MICS, including early chest tube removal, led to earlier intensive care unit discharge, reduced hospital length of stay, and improved patient-reported outcomes.^39^ These findings align with other studies emphasizing the importance of chest tube removal to facilitate early ambulation and pulmonary rehabilitation. Institutional protocols should include clear criteria for early drain removal, such as minimal output, absence of air leak, and stable hemodynamics. Multidisciplinary collaboration among surgeons, critical care physicians, nursing staff, respiratory therapists, and other members of the health care team is essential to implement these protocols in a safe and effective manner.

## 6. Measure Functional Pain

Self-reported numerical rating scales (NRS) record pain intensity at the moment of assessment, whereas morphine milligram equivalent (MME) quantifies opioid utilization. NRS and MME are frequently used as outcomes in pain research and clinical quality improvement initiatives because of their familiarity and ease of administration. However, both fail to capture the effect that pain has on the achievement of recovery milestones such as deep breathing, coughing, eating, drinking, and mobilizing.^40^ Although associated, they imperfectly reflect the quality of recovery from the patient’s perspective.^41–43^ Treatment focused on NRS alone may expose patients to analgesic-related side effects without necessarily improving recovery outcomes. Finally, MME does not reflect individual variations in metabolism, pain sensitivity, or subjective pain experience.^44,45^ Functional pain measures are superior because they evaluate the impact of pain on postoperative recovery and patient experience.^43^ Ideal tools are validated multidimensional instruments that quantify pain intensity with activity, pain-limited functional capacity, patient satisfaction, and achievement of recovery milestones. Several tools have been previously described, with varying elements of emphasis and durations of completion (Table 2).^46–51^

**Table 2.** Summary of Options for Functional Pain Assessment Tools.

Tool	Description	Validation population	Time to administer
Brief Pain Inventory (BPI)^46^	Pain intensity and interference with daily activities	Surgical population	SF 5 min LF 10 min
McGill Pain Questionnaire (MPQ2)^47^	Quantitative and qualitative measurement of pain	Widely validated	SF 5 min LF 10–15 min
Prince Henry Hospital Pain Score (PHHPS)^48^	Pain intensity on a 0–4 scale with essential recovery activities including rest, deep breathing, coughing, and movement	Thoracic and cardiac surgical patients	<5 min
Quality of recovery 15 (QoR-15)^49^	Quality of recovery domains including pain, mobility, and general well-being	Surgical	<5 min
Johns Hopkins Highest Level of Mobility (JH-HLM)^50^	Highest level of mobility on an 8-point scale	Surgical and critical care populations	<5 min
Functional activity scale (FAS)^51^	Impact of pain on a single selected activity is graded on a 3-point scale	Surgical	<5 min

Abbreviations: LF, long form; SF, short form.

## 7. Ongoing Audit of Analgesia Effectiveness

Audit is a crucial component of quality improvement and enhanced recovery programs.^52,53^ Properly designed, implemented, and disseminated audit programs can lead to sustained benefits for patients and health care systems through multiple mechanisms. It can help create awareness among the perioperative team, foster a sense of urgency toward supporting improvements, facilitate knowledge sharing within different disciplines, and help eliminate perceived hierarchies, which encourages collaboration and honest feedback.^54^ A comprehensive analgesia audit plan within an MICS program will allow tracking compliance with care elements that reduce pain, determine the effectiveness of current strategies, measure pain and analgesic-related adverse effects, and reflect the patient’s quality of recovery.^55,56^
